# Transcriptional Patterns of Nodal Entropy Abnormalities in Major Depressive Disorder Patients with and without Suicidal Ideation

**DOI:** 10.34133/research.0659

**Published:** 2025-04-02

**Authors:** Minxin Guo, Heng Zhang, Yuanyuan Huang, Yunheng Diao, Wei Wang, Zhaobo Li, Shixuan Feng, Jing Zhou, Yuping Ning, Fengchun Wu, Kai Wu

**Affiliations:** ^1^ School of Biomedical Sciences and Engineering, South China University of Technology, Guangzhou International Campus, Guangzhou, China.; ^2^ Department of Psychiatry, The Affiliated Brain Hospital, Guangzhou Medical University, Guangzhou, China.; ^3^ School of Material Science and Engineering, South China University of Technology, Guangzhou, China.; ^4^ National Engineering Research Center for Tissue Restoration and Reconstruction, South China University of Technology, Guangzhou, China.; ^5^ Guangdong Engineering Technology Research Center for Translational Medicine of Mental Disorders, Guangzhou, China.; ^6^ Key Laboratory of Neurogenetics and Channelopathies of Guangdong Province and the Ministry of Education of China, Guangzhou Medical University, Guangzhou, China.; ^7^ Department of Aging Research and Geriatric Medicine, Institute of Development, Aging and Cancer, Tohoku University, Sendai, Japan.

## Abstract

Previous studies have indicated that major depressive disorder (MDD) patients with suicidal ideation (SI) present abnormal functional connectivity (FC) and network organization in node-centric brain networks, ignoring the interactions among FCs. Whether the abnormalities of edge interactions affect the emergence of SI and are related to the gene expression remains largely unknown. In this study, resting-state functional magnetic resonance imaging (fMRI) data were collected from 90 first-episode, drug-naive MDD with suicidal ideation (MDDSI) patients, 60 first-episode, drug-naive MDD without suicidal ideation (MDDNSI) patients, and 98 healthy controls (HCs). We applied the methodology of edge-centric network analysis to construct the functional brain networks and calculate the nodal entropy. Furthermore, we examined the relationships between nodal entropy alterations and gene expression. The MDDSI group exhibited significantly lower subnetwork entropy in the dorsal attention network (DAN) and significantly greater subnetwork entropy in the default mode network than the MDDNSI group. The visual learning score of the measurement and treatment research to improve cognition in schizophrenia (MATRICS) consensus cognitive battery was negatively correlated with the subnetwork entropy of DAN in the MDDSI group. The support vector machine model based on nodal entropy achieved an accuracy of 81.87% when distinguishing the MDDNSI and MDDSI. Additionally, the changes in SI-related nodal entropy were associated with the expression of genes in cell signaling and interactions, as well as immune and inflammatory responses. These findings reveal the abnormalities in nodal entropy between the MDDSI and MDDNSI groups, demonstrated their association with molecular functions, and provided novel insights into the neurobiological underpinnings and potential markers for the prediction and prevention of suicide.

## Introduction

Major depressive disorder (MDD) is a severe psychiatric disorder that is characterized by a high prevalence, a high recurrence rate, and a high suicide rate [1]. Suicidal ideation (SI) is considered a high-risk factor for suicidal behaviors in MDD patients [2,3]. More than half of the MDD patients experienced SI, and among those who attempted suicide, approximately 95% reported having experienced SI prior to their attempts [4,5]. Identifying individuals with MDD with SI (MDDSI) is a substantial public health challenge and has marked clinical implications [6]. In addition, MDD patients are associated with cognitive impairments and exhibit deficits in attention, memory, and reward processing [7]. Importantly, the cognitive performance of MDDSI patients is closely impacted by the severity of SI [8]. However, the relationship between altered brain activity and cognitive ability in MDDSI patients remains uncertain.

To improve the prevention and intervention of MDDSI, numerous neuroimaging studies have explored the complex biosignatures of MDDSI [9]. Specifically, MDDSI patients exhibit substantial functional deficits in the prefrontal cortex, temporal lobe, and limbic system, areas that are crucial for emotional regulation and cognitive control [10,11]. Additionally, several previous studies have shown that MDDSI patients have decreased resting-state functional connectivity (RSFC) among the left frontoparietal network, anterior default mode network, and salience network compared with patients with MDD without SI (MDDNSI), and the decreased RSFC of salience network was correlated with the severity of SI in MDD patients [12–14]. However, most studies have focused on nodes by quantifying their degree, centrality, or communities to investigate the emergence of SI in MDD patients and ignored the descriptions and implications of edge interactions [15]. Recently, Faskowitz et al. [16] proposed a new method to investigate edge interactions from the perspective of an edge-centric network. This methodology introduces an “unfold” Pearson correlation coefficient to calculate the functional connectivity (FC) between pairs of brain regions, leading to the generation of edge community structures via K-means clustering algorithms [16]. Importantly, when the FC between pairs of brain regions maps to other brain regions, these edge communities can form an overlapping function and structure, measured by nodal entropy [17]. Recently, the analysis of edge-centric networks has been applied in the study of neuropsychiatric disorders [18]. Specifically, patients with autism spectrum disorder exhibit abnormal co-fluctuations among the amygdala, pallidum, hippocampus, and thalamus [18]. Additionally, the nodal entropy of the whole brain of stroke patients is correlated with the severity of stroke lesions and continually increases over the course of patient recovery [19]. Thus, edge-centric network analysis has the potential to explore the neuropathology of functional impairments in first-episode drug-naive MDD patients with and without SI.

The advent of whole-brain gene expression atlases has revolutionized the exploration of the intricate relationships between macrolevel brain network alterations and microlevel gene expression patterns [20,21]. The integration of connectomics, transcriptomics, and genetics has provided new insights into the neurobiological underpinnings of psychiatric disorders such as schizophrenia and MDD [22,23]. In addition, this approach can reveal the multifaceted nature of MDD and emphasize the critical role of transcriptional signatures in both research methodologies and the search for mechanisms [24,25]. Therefore, the analysis of the genetic data associated with the nodal entropy of edge-centric networks can reveal the neural pathway implicated in the predisposition to suicide in MDD patients.

**Fig. 1.**
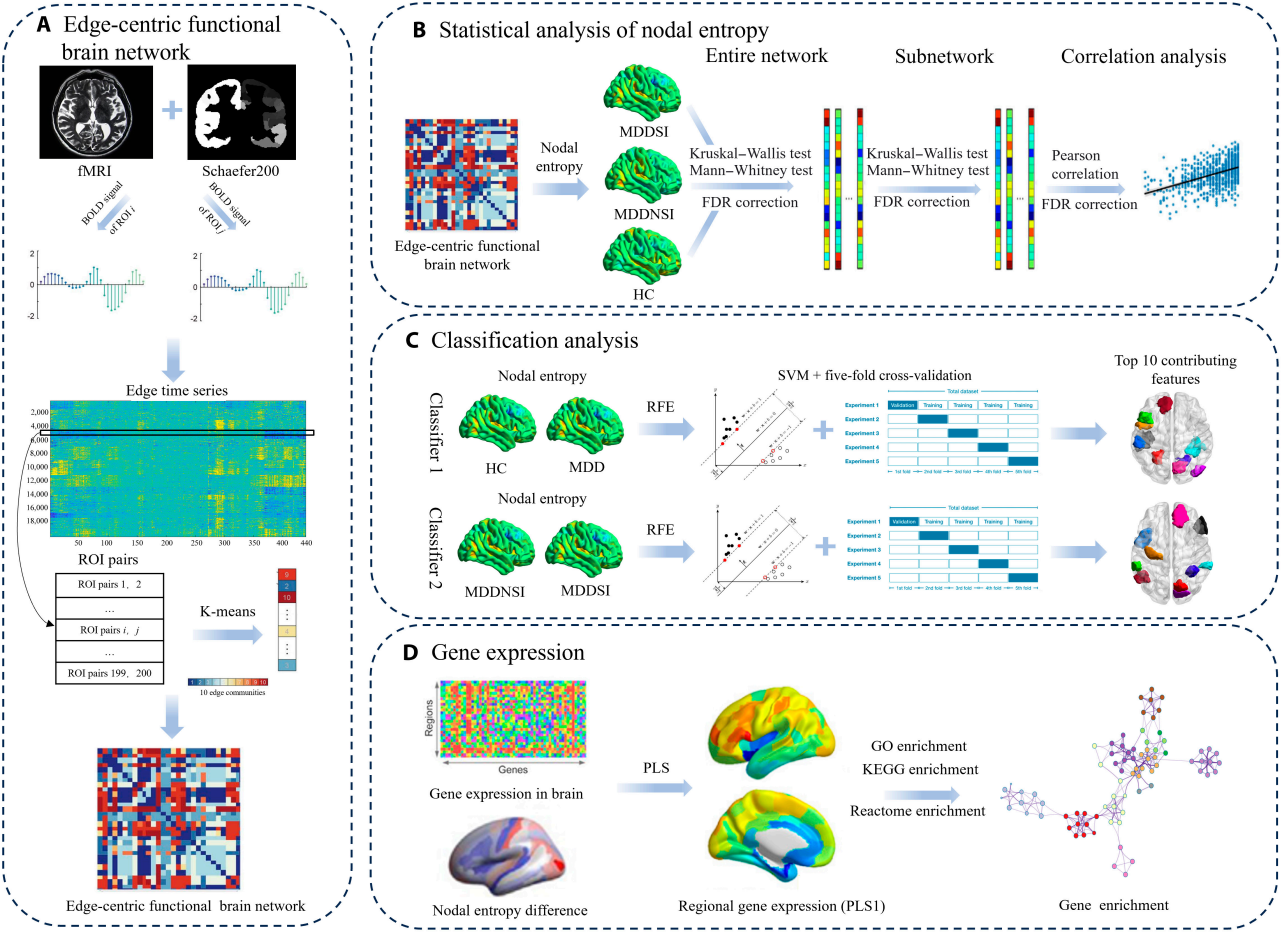
Overview of the analysis pipeline. (A) Construction of the edge-centric brain network. (B) Statistical analysis of nodal entropy. (C) Classification analysis based on the nodal entropy. (D) Gene expression data processing.

In this study, we aimed to analyze edge-centric functional brain network (FBN) to investigate the neural edge interactions in first-episode drug-naive MDDSI patients and explore related transcriptional profiles (Fig. 1). We first examined the nodal entropy differences among the healthy controls (HCs), MDDNSI, and MDDSI groups and analyzed the correlation between nodal entropy and clinical scale scores. Second, we explored the diagnostic value of nodal entropy in identifying MDD and MDDSI using support vector machines (SVMs). Third, we used the gene expression data from the Allen Human Brain Atlas to probe the connectome and transcriptome association patterns. We hypothesized that (a) there would be distinct differences in nodal entropy between MDDSI and MDDNSI groups, and that (b) alterations in SI-related nodal entropy would be associated with gene expression profiles enriched in biologically pathways.

## Results

### Demographic characteristics

According to the inclusion criteria, the final sample for subsequent analysis consisted of 90 MDDSI, 60 MDDNSI, and 98 HCs. The demographic and clinical assessments for each group are described in Table 1. In particular, demographically, the HCs, MDDNSI, and MDDSI groups significantly differed in age, sex, and years of education. These differences should be considered covariates to eliminate potential confounding effects. In addition, the scores in all sections of the measurement and treatment research to improve cognition in schizophrenia (MATRICS) consensus cognitive battery (MCCB) were significantly different among the HCs, MDDNSI, and MDDSI groups. The total scores of the Hamilton Depression Scale (HAMA)-17 and the third item scores of the HAMA-17 in the MDDSI group were significantly higher than those in the MDDNSI group. Additionally, we performed post-hoc tests among the MDDSI, MDDNSI, and HCs, in terms of sex, age, years of education, 5 dimension scores of MCCB, and the total scores of MCCB, as shown in Table 2. The 5 dimension scores of MCCB and the total scores of MCCB in the HCs group were significantly higher than those in both the MDDSI and MDDNSI groups. The age in the HCs group was significantly lower than those in both the MDDSI and MDDNSI groups, and the age in the MDDNSI group was significantly lower than those in the MDDSI group. The years of education in the HCs group was significantly higher than those in both the MDDSI and MDDNSI groups, and the years of education in the MDDNSI group was significantly higher than those in the MDDSI group. In addition, the sex differences were significant between the HCs and MDDSI groups, as well as between HCs and MDDNSI groups.

**Table 1.** Group demographics of our recruited subjects

Characteristics	HCs group (*n* = 98)	MDDNSI group (*n* = 60)	MDDSI group (*n* = 90)	Group comparison
Sex (female/male)	49 females, 49 males	41 females, 19 males	60 females, 30 males	*χ*^2^ = 7*.*49, *P* = 0*.*024^a^
Age (years), mean (SD)	22.00 (2.62)	25.00 (4.94)	22.00 (2.62)	*F* = 10*.*31, *P* < 0*.*001^b^
Years of education, mean (SD)	13.82 (2.51)	14.85 (2.69)	16.00 (2.05)	*F* = 15*.*63, *P* < 0*.*001^b^
The total scores of HAMD-17, mean (SD)	–	21.08 (4.74)	23.69 (2.16)	*t* = 11.94, *P* < 0.001^c^
The third item scores of HAMD-17, mean (SD)	–	0.93 (0.98)	2.11 (1.04)	*t* = 6.87, *P* < 0.001^c^
MCCB speed of processing, mean (SD)	45.61 (9.90)	33.52 (10.60)	31.33 (10.50)	*F* = 50*.*46, *P* < 0*.*001^b^
Attention/Vigilance, mean (SD)	42.24 (8.53)	34.12 (10.78)	33.60 (10.26)	*F* = 21*.*98, *P* < 0*.*001^b^
Working memory, mean (SD)	46.99 (10.68)	39.20 (11.29)	37.87 (10.62)	*F* = 18*.*76, *P* < 0*.*001^b^
Verbal learning, mean (SD)	41.62 (7.99)	32.58 (10.28)	32.78 (10.87)	*F* = 24*.*86, *P* < 0*.*001^b^
Visual learning, mean (SD)	45.29 (7.08)	39.97 (7.99)	40.59 (8.64)	*F* = 11*.*60, *P* < 0*.*001^b^
Total score, mean (SD)	221.76 (30.54)	179.38 (50.93)	176.17 (50.91)	*F* = 49*.*33, *P* < 0*.*001^b^

^a^ Chi-square test.

^b^ One-way ANOVA.

^c^ Two-sample *t* tests.

**Table 2.** The post-hoc comparisons results of demographics and clinical scale

	MDDSI-MDDNSI	MDDNSI-HCs	MDDSI-HCs
Sex	*P* = 0.831	*P* = 0.0315	*P* = 0.012
Age	*P* < 0.001	*P* < 0.001	*P* < 0.001
Years of education	*P* = 0.011	*P* = 0.018	*P* < 0.001
Speed of processing	*P* = 0.207	*P* < 0.001	*P* < 0.001
Attention/Vigilance	*P* = 0.752	*P* < 0.001	*P* < 0.001
Working memory	*P* = 0.463	*P* < 0.001	*P* < 0.001
Verbal learning	*P* = 0.905	*P* < 0.001	*P* < 0.001
Visual learning	*P* = 0.639	*P* < 0.001	*P* < 0.001
The total of MCCB	*P* = 0.575	*P* < 0.001	*P* < 0.001

### Differences in nodal entropy across the entire network and subnetworks

**Fig. 2.**
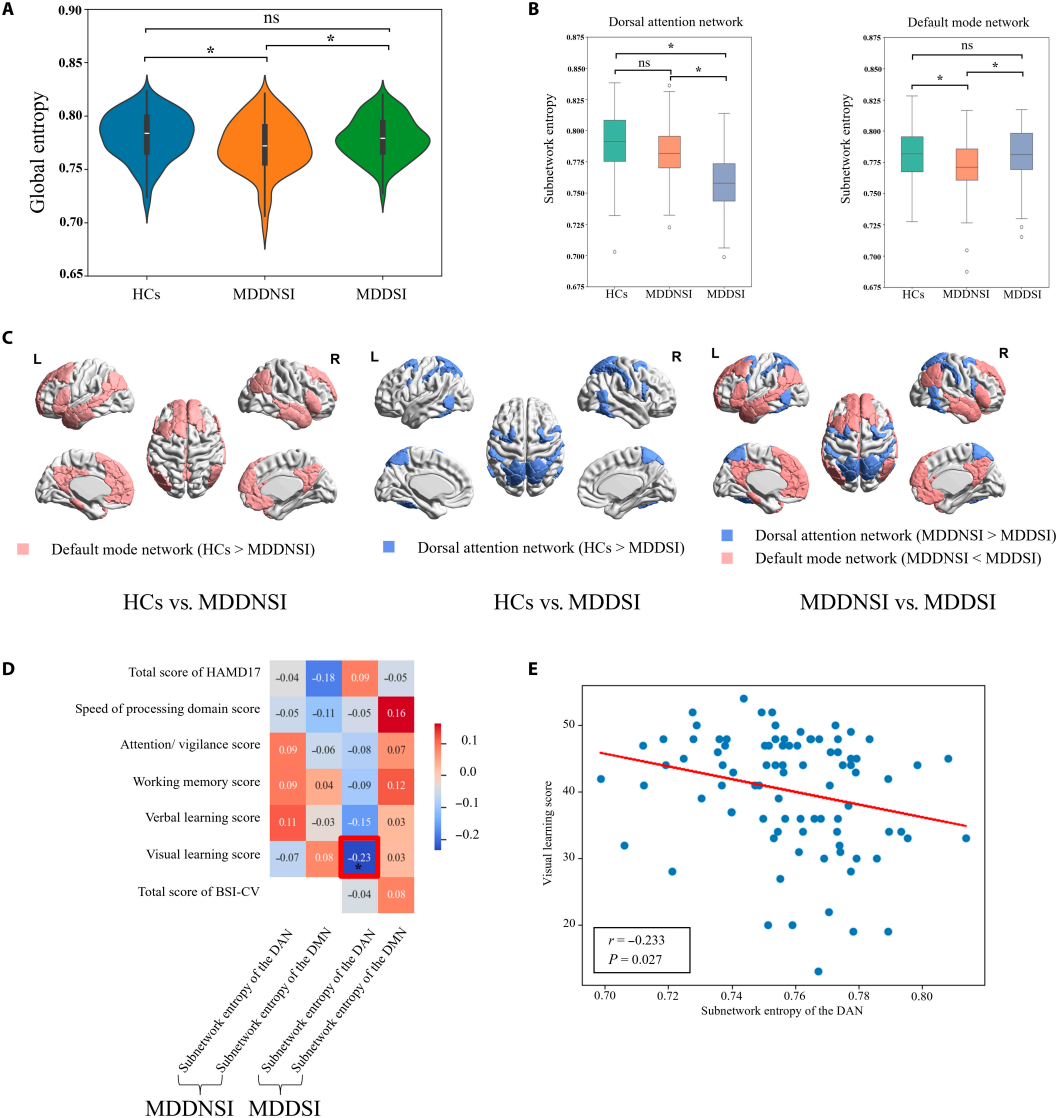
Differences in global entropy and nodal entropy of subnetworks. (A) Differences in global entropy among HCs, MDDNSI, and MDDSI groups. (B) Differences in nodal entropy of subnetworks among HCs, MDDNSI, and MDDSI groups. (C) Visualizations of significant different subnetworks. (D) Correlation between nodal entropy of the significantly different subnetwork and clinical scale scores. (E) Correlations between nodal entropy of the subnetwork of the DAN and visual learning score in the MDDSI.

We found significant between-group differences in global entropy (defined as the mean nodal entropy across the entire network) between the MDDSI and MDDNSI groups and between the HCs and MDDNSI groups [*P <* 0*.*05, false discovery rate (FDR) corrected]. Moreover, the global entropy in the HCs group was significantly higher than that in the MDDNSI group. With the emergence of SI in MDD patients, the global entropy in the MDDSI group was significantly higher than that in the MDDNSI group (Fig. 2A).

Additionally, the subnetwork entropy (defined as the mean nodal entropy across the subnetwork) of the dorsal attention network (DAN) in the MDDSI groups was significantly lower than that in the HCs and MDDNSI groups (*P <* 0*.*05, FDR corrected). The subnetwork entropy of the default mode network (DMN) in the MDDSI group was significantly higher than that in the MDDNSI group (*P <* 0*.*05, FDR corrected). The subnetwork entropy of the DMN in the MDDNSI group was significantly lower than that in the HCs group (*P <* 0*.*05, FDR corrected) (Fig. 2B). In Fig. 2C, we presented visualizations of specific brain regions between the 2 groups among these 3 groups.

Moreover, in the MDDSI group, the Pearson correlation analysis revealed that the visual learning score of the MCCB was negatively correlated with the subnetwork entropy of the DAN (*r* = −0*.*233, *P* = 0*.*027, FDR corrected) (Fig. 2E). Additionally, in the MDDNSI group, no significant correlation was found between subnetwork entropy and either the total or 5 dimension scores of MCCB, or the total score of HAMD-17 (Fig. 2D).

### Classification performance based on nodal entropy

**Fig. 3.**
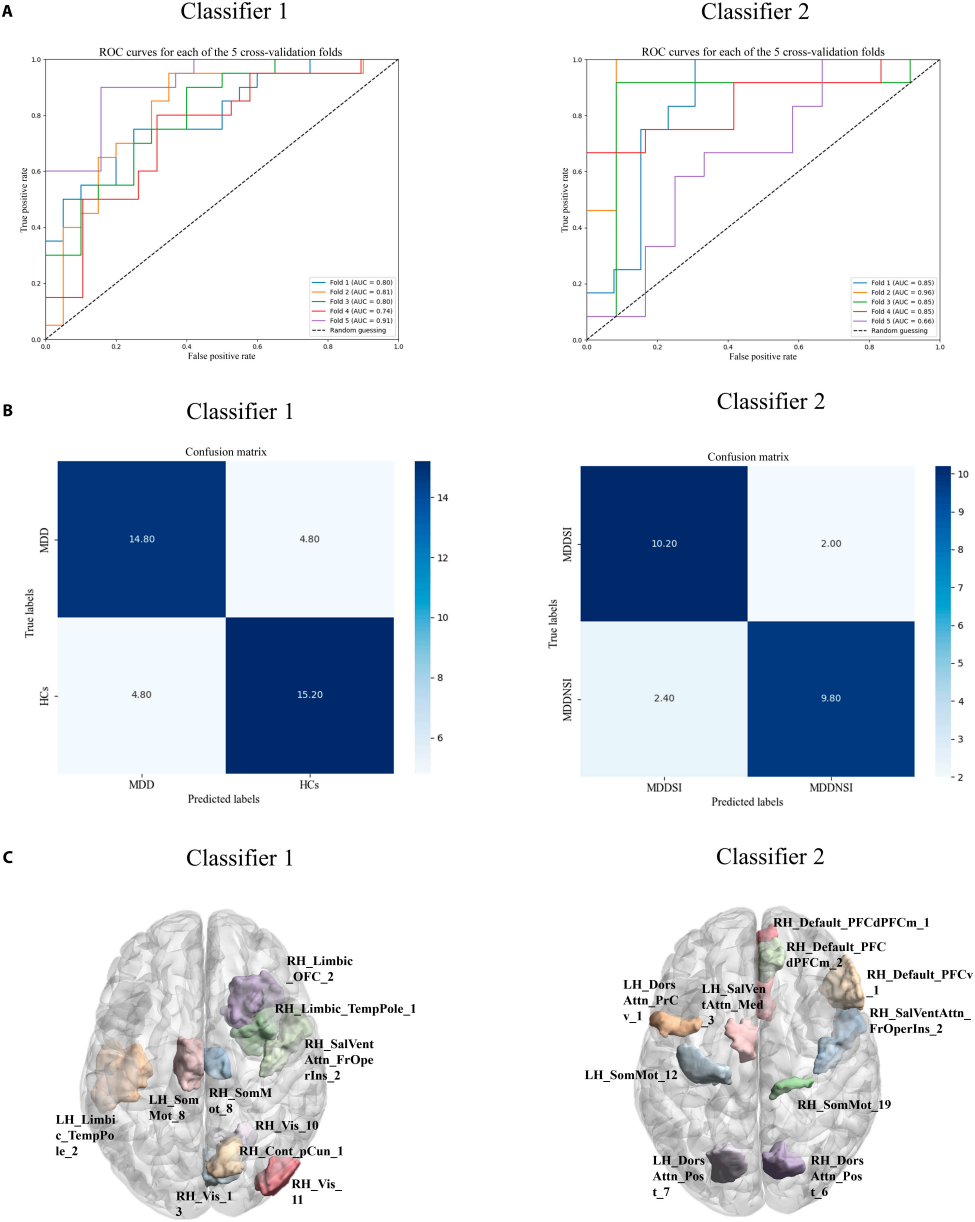
Classification performance of classifier 1 and classifier 2 based on nodal entropy. (A). Receiver operating characteristic curves of 5 cross-validation folds for classifier 1 and classifier 2 (B) Confusion matrices for classifier 1 and classifier 2. (C) Top 10 contributing features for classifier 1 and classifier 2.

We developed 2 classifiers to distinguish between HCs and MDD patients and between MDDSI patients and MDDNSI patients using the linear SVM model, respectively. We used the recursive feature elimination (RFE) method to perform feature selection based on the nodal entropy. For classifier 1 and classifier 2, we selected 60 features for classification (Tables S1 and S2). For classifier 1, using 5-fold cross-validation, we achieved an average accuracy of 75*.*78%, an average precision of 76*.*69%, an average recall of 76*.*00%, and an optimal F1 score of 75*.*93% (Fig. 3A and B). For classifier 2, under 5-fold cross-validation, we achieved an average accuracy of 81*.*87%, an average precision of 83*.*20%, an average recall of 80*.*00%, and an average F1 score of 80*.*91% (Fig. 3A and B). The top 10 contributing features for classifier 1 were primarily from the visual network and limbic system (Fig. 3C and Table S4). The top 10 contributing features for classifier 2 were primarily from the DMN and DAN (Fig. 3C and Table S5).

### Transcriptional profiles associated with SI

**Fig. 4.**
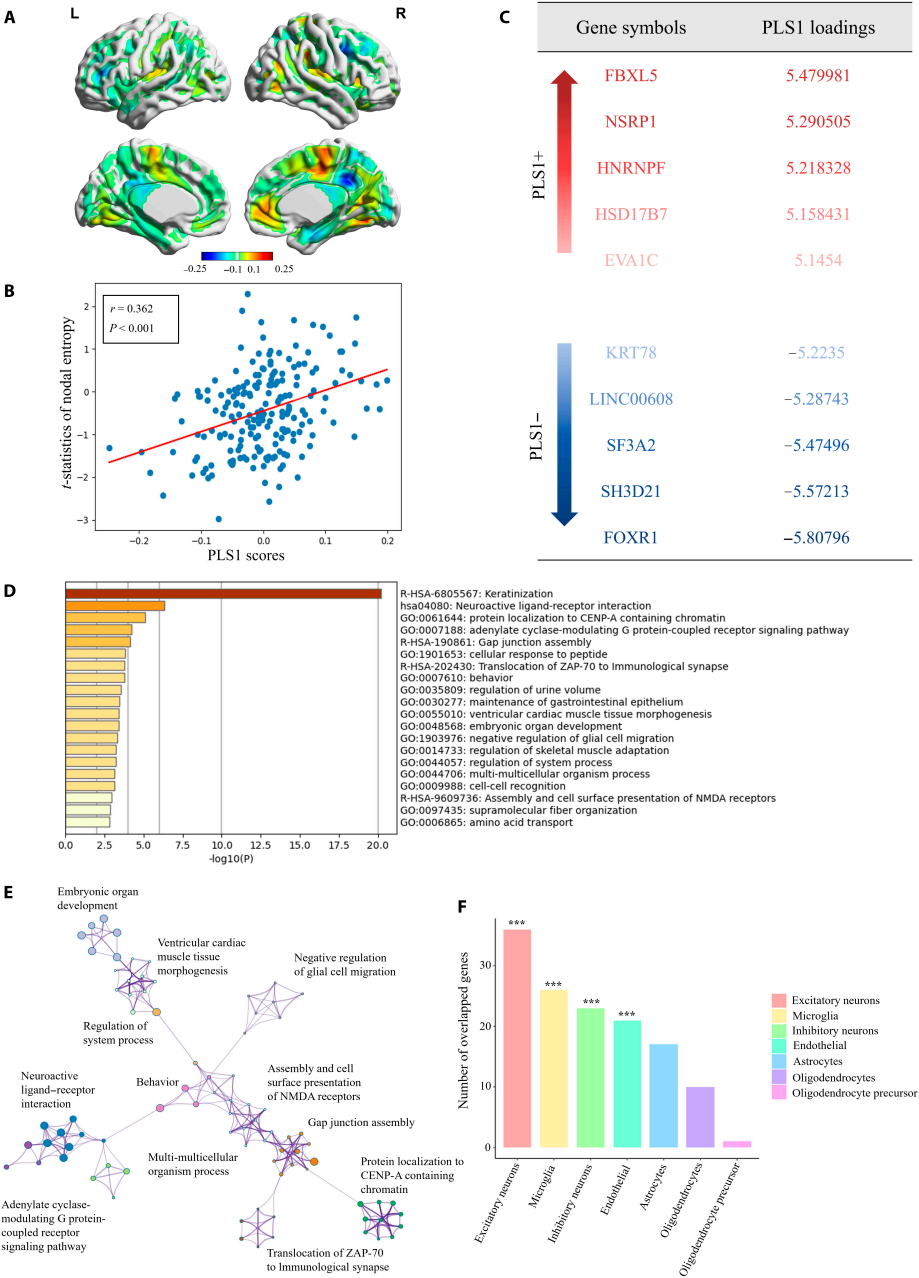
Transcriptional profiles associated with SI-related nodal entropy changes. (A) Brain map of the first component of partial least square (PLS1) scores. (B) Correlations between PLS1 score and nodal entropy changes between MDDSI and MDDNSI. (C) List of the PLS1+ and PLS1− genes. (D) Enrichment of the PLS1− genes. (E) Enrichment network of the intra- and intercluster similarities of enriched annotations. (F) Number of significantly PLS1− genes that overlap with gene sets for each cell type.

PLS1 accounted for 14% of the variance in the SI-related nodal entropy changes, which were subsequently examined for spatial associations with gene transcriptional profiles (*P <* 0*.*001) (Fig. 4A and Fig. S1). We found a spatial correlation between the PLS1 score map and the *t*-statistics maps of nodal entropy (*r* = −0*.*362, *P <* 0*.*0001, FDR corrected) (Fig. 4B). By ranking 15,632 genes according to their corrected weights, we identified 788 genes with strongly positive PLS1 weights (referred to as the PLS1+ gene set) and 900 genes with strongly negative PLS1 weights (referred to as the PLS1− gene set) (Fig. 4C).

The enrichment analysis revealed a notable increase in the PLS1− gene set across various clusters, notably in the Kyoto Encyclopedia of Genes and Genomes (KEGG), Gene Ontology (GO), and Reactome pathways (*P <* 0*.*05, FDR corrected). After discarding discrete clusters, the top enrichment terms were related mainly to the processes of cell signaling, cell interaction, immune responses, and inflammatory responses (Fig. 4D and E). The PLS1+ gene set showed no significant clusters of enrichment. Based on the distribution of genes across 7 standard cell types, there was a significant correlation between PLS1− genes and excitatory neurons (*n* = 36, *P <* 0*.*001, FDR corrected), microglia (*n* = 26, *P <* 0*.*001, FDR corrected), inhibitory neurons (*n* = 23, *P <* 0*.*001, FDR corrected), and endothelial cells (*n* = 21, *P <* 0*.*001, FDR corrected) (Fig. 4F).

## Discussion

To our knowledge, this is the first study to examine the nodal entropy of the MDDNSI and MDDSI using an edge-centric network analysis. Our main findings indicated that the MDDSI group exhibited (a) significantly greater global entropy and the subnetwork entropy of the DMN, and significantly lower subnetwork entropy of the DAN than the MDDNSI group; (b) a significant negative correlation between the visual learning score of the MCCB and the subnetwork entropy of the DAN; and (c) SI-related nodal entropy changes associated with biological processes, such as cell signaling and interaction, and immune and inflammatory responses.

### Alterations in global entropy and subnetwork entropy

In this study, we calculated the nodal entropy for each brain region from the edge-centric FBN. An increase in nodal entropy reflects an increase in the activity level of the overlapping community of brain regions within the edge-centric FBN, leading to increased functional overlap between regions [16]. Therefore, an increase in nodal entropy implies that brain regions or subnetworks become more functionally segregated; nevertheless, a decrease in nodal entropy implies that brain regions or subnetworks become more functionally integrated [17].

Our results demonstrated that the global entropy in the MDDNSI group was significantly lower than that in the HCs group. The brain regions or subnetworks might become more functionally integrated in the MDDNSI group [26]. We speculated that the decrease in global entropy in the MDDNSI group might be associated with deficits in functional modules and organization [27,28]. Moreover, the global entropy in the MDDSI group was significantly higher than that in the MDDNSI group. The finding suggested that the presence of SI in the MDDSI group is associated with an increase in the overlap of functional modules between brain regions, resulting in a more functionally segregated and active state of the overlapping community of the brain [29].

In this study, we also found that the subnetwork entropy of the DAN in the MDDSI group was significantly lower than those in the MDDNSI and HC groups. This finding indicated that the DAN becomes more integrated in the MDDSI group, reflecting substantial functional disruption within the DAN. The DAN is responsible for the bottom-up regulation of visual objects and top-down attentional selection [30,31]. Importantly, MDD patients with SI exhibit excessive self-referential processing, while significantly impairing attentional capacity for external stimuli (top-down regulation). Thus, our findings may provide a novel perspective for understanding the mechanism of MDD patients with SI.

In addition, we observed that the subnetwork entropy of the DMN in the MDDNSI group was significantly lower than that in the HCs group. The DMN plays an important role in the psychological processes associated with suicide, and the DMN-related regions are sensitive to internal information, such as beliefs, emotions, and long-term memory, and control the vividness of negative mental images and improve self-referential processing [32,33]. Nevertheless, the subnetwork entropy of the DMN in the MDDSI group was significantly higher than that in the MDDNSI group. This finding indicated that the DMN became more segregated in the MDDSI group. The SI is closely associated with negative emotions and thoughts in MDD patients, known as negative rumination, which may result in the increase of functional activity in the DMN [34,35].

Furthermore, there was no significant differences in the global entropy and the subnetwork entropy of the DMN between the MDDSI and HCs groups. We speculated that the absence of significant differences in global entropy and subnetwork entropy of the DMN might be attributed to a compensatory mechanism within both the whole brain and the DMN, accompanied with substantial functional disruption within the DAN [36]. A previous study has identified the DAN and DMN as typically anticorrelated networks [37]. Thus, our findings suggested that the reduction in the subnetwork entropy of the DAN may be associated with a relative increase in both the subnetwork entropy of the DMN and the global entropy.

### Cognitive implications

In this study, our results indicated a significantly negative correlation between the subnetwork entropy of the DAN and visual learning scores exclusively in the MDDSI group. Our previous study has demonstrated that the functional activity of precuneus, the core of the DMN, is positively correlated with visual learning scores in bipolar patients with SI [38]. These findings suggested that both the DAN and the DMN are critical subnetworks associated with visual learning ability. More importantly, the DAN and the DMN typically show anticorrelated activity in the spontaneous neural activity during rest [39]. Therefore, our findings implied that the reduced subnetwork entropy of the DAN in MDDSI patients may result from a relative increase in the subnetwork entropy of the DMN, which maintain relatively higher visual learning performance in the MDDSI group.

### Classification performance of nodal entropy

Individual-level classification performance highlighted the diagnostic value of nodal entropy for both MDD identification and SI detection. Our study achieved an accuracy of 75.75% for the classification between HCs and MDD groups and an accuracy of 81.67% for the classification of MDDNSI and MDDSI groups. Previously, Huang et al. [40] have utilized the amplitude of low-frequency fluctuations to classify MDD patients and HCs, achieving an accuracy of 81.9%. Similarly, Xu et al. [41] have used dynamic functional networks to classify the MDDSI and MDDNSI groups, reporting an accuracy of 75%. In addition, deep learning has also been applied in MDD identification and SI detection. Zhou et al. [42] have employed a dynamic graph convolutional network to classify MDD and HC groups, achieving an accuracy of 82.5%, while Hu et al. [43] have used a fully connected neural network to classify MDDSI and MDDNSI groups, achieving an accuracy of 70.12%. Interestingly, in this study, the classification between MDDSI and MDDNSI groups revealed that the important features were primarily from the DAN and DMN. Nevertheless, in this study, we did not collect genetic data, and thus no genetic data were available for analyzing MDD and MDDSI. However, prior research has demonstrated the feasibility of using genetic and magnetic resonance imaging (MRI) data for MDD classification [44]. Additionally, previous studies have shown that combining genetic and neuroimaging data could enhance classification performance [45]. In the future study, we can adopt multimodal approaches that integrate genetic markers and imaging features, providing deep insights into the neurobiological basis of MDD and MDDSI.

### Connectome and transcriptome association analysis

The analysis of the relationship between the connectome and transcriptome revealed a link between SI-related nodal entropy changes and gene expression related to cell signaling and interactions, as well as immune and inflammatory responses. Postmortem brain tissue studies have identified alterations in gene expression related to metabolism, transcription, and neurotransmission, which are important for understanding the neurobiology of MDDSI [46]. Cell signaling and interactions and immune and inflammatory responses are crucial biological processes connected to synaptic plasticity [47]. Decreased neuroplasticity has been associated with impaired stress coping mechanisms and may play a role in the development of SI in individuals with MDD [48]. Previous research also suggested that the regular intake of nutrients that support neurotransmission might decrease the suicide risk and increase the effects of psychotropic medications [49].

We divided the SI-related genes into specific cell types based on each cellular gene set. These genes were notably linked to excitatory neurons, microglia, inhibitory neurons, endothelial cells, and astrocytes. Impairments in glutamatergic excitatory neurons may result in reduced levels of serotonin and brain-derived neurotrophic factor, both of which are linked to the neurobiological mechanisms of suicide [50]. Microglia, which are immune cells of the brain, play a vital role in the development of suicide risk factors, including childhood trauma and stress. Abnormalities in inhibitory neurons can lead to dysfunction of the gamma-aminobutyric acid system, which may trigger negative thinking patterns and emotional disorders, all of which are potential factors for suicide risk [51]. Previous studies have suggested that MDD patients may experience endothelial cell dysfunction. This dysfunction indirectly increases the risk of suicide by impairing cerebral blood flow and exacerbating neuroinflammation [52].

## Conclusion

In conclusion, the MDDSI group presented significant differences in the global entropy and subnetwork entropy of the DAN and DMN from those of the MDDNSI group, and there was a significant negative correlation between the visual learning score of the MCCB and the subnetwork entropy of the DAN. Nodal entropy is considered a biomarker for identifying MDD and MDDSI at the individual level. Furthermore, changes in SI-related nodal entropy are associated with biological processes, such as cell signaling and interactions, as well as immune and inflammatory responses. The findings of this study improve the understanding of the emergence of SI in MDD patients. Distinct abnormalities in nodal entropy may mediate the progression of SI in MDD patients and can serve as a reliable biomarker for auxiliary diagnosis, particularly in those with or without SI.

## Materials and Methods

### Participants

This study included 163 first-episode, drug-naive MDD patients and 98 HCs. All participants provided informed consent forms after comprehending the study procedure. The study received approval from the Ethics Committee of the Affiliated Brain Hospital of Guangzhou Medical University (ethical approval number: AF/SC-08/02.3) and adhered to the most recent Declaration of Helsinki guidelines (2013). Diagnoses of first-episode, drug-naive MDD were established by a trained clinician, who conducted a structured clinical interview with all participants on the basis of the *Diagnostic and Statistical Manual of Mental Disorders, Fifth Edition* (*DSM-V*). We subsequently screened 150 first-episode, drug-naive MDD patients and 98 HCs by excluding the subjects without the Chinese adaptation of the Beck Scale for Suicide Ideation (BSI-CV) complete scores.

The inclusion criteria for all patients were as follows: (a) patients with depression who met the *DSM-V* diagnostic classification criteria for MDD; (b) ages ranging from 15 to 40 years; (c) patients with first-episode MDD, with a disease duration of not >2 years [53–56]; and (d) no medication history. The HCs were recruited from the general population via advertisements. HCs included any past or present psychiatric conditions, such as depressive disorder, bipolar disorder, or substance abuse/dependence, or those with current or past serious medical and neurological disorders were excluded.

In this study, the BSI-CV was administered to assess the severity of suicidal thoughts, behaviors, and plans in patients within the past week. In previous suicide studies, the BSI-CV scale has been shown to be more targeted and sensitive, providing a more accurate reflection of the specific characteristics of suicidal tendencies [57]. In this study, SI was identified by a score of higher than or equal to 1 on item 4 or 5 of the BSI-CV (participants who indicated weak or moderate to strong on these items were classified as having SI) [58]. Based on the presence or absence of SI, first-episode drug-naive MDD patients were separated into 2 groups: the group with SI was referred to as the MDDSI group (*n* = 90), and the group without SI was referred to as the MDDNSI group (*n* = 60).

### Cognitive assessment scale

The cognitive performance of all the participants was assessed using the Chinese version of the Measurement and Treatment Research to Improve Cognition in Schizophrenia Consensus Cognitive Battery (MCCB), which has been shown to have good reliability in the Chinese population [59]. The MCCB, developed by the MATRICS group, includes 10 tests selected from over 90 options to assess cognitive impairment in patients with schizophrenia and mood disorders. It has been translated into several languages and is widely used [60]. In this study, we included only the 5 dimensions of the Chinese version of the MCCB, which were used in our previous studies [38,54,61–63]: speed of processing (SOP), attention/vigilance (AV), working memory (WM), verbal learning (VRB), and visual learning (VIS). SOP includes the Trail Making Test-A, Category Fluency, and Brief Assessment of Cognition in Schizophrenia; AV is assessed by the Continuous Performance Test-Identical Pairs version; WM is measured using the Wechsler Memory Scale-Spatial Span subtest; VRB is assessed via the Hopkins Verbal Learning Test-Revised; and VIS is evaluated via the Brief Visuospatial Memory Test-Revised. All tests were administered by trained psychiatrists or graduate students, and all research staff underwent a training session. The interrater reliability was above the critical value of 0.8 [64].

### MRI data acquisition and preprocessing

A Siemens Prisma 3.0 T scanner with system version E11 was used to acquire MRI images from all participants at the Affiliated Brain Hospital of Guangzhou Medical University. The multi-band technique was employed in the EPI scan, achieving a repetition time (TR) of 800 ms with 450 time points (repetitions) over a total duration of 6 min for the resting-state functional magnetic resonance imaging (fMRI) (rs-fMRI) scan. For all participants, rs-fMRI data were obtained with the following parameters. TR = 800 ms, echo time (TE) = 30 ms, field of view (FOV) = 208 mm × 208 mm, flip angle (FA) = 56°, acquisition resolution = 2 mm × 2 mm × 2 mm, matrix = 104 × 104, multiband acceleration factor = 8, slice thickness = 2 mm, spacing between slices = 0 mm, number of slices = 72, and pixel bandwidth = 2,290 Hz. Structural MRI data were obtained via magnetization-prepared rapid acquisition gradient echo sequences with the following parameters. TR = 2,000 ms, TE = 2.32 ms, time inversion = 900 ms, FOV = 230 mm × 230 mm, FA = 8°, acquisition resolution = 0.9 mm × 0.9 mm × 0.9 mm, matrix = 256 × 256, slice thickness = 0.9 mm, number of slices = 208, parallel reduction factor in plane = 2, and pixel bandwidth = 200 Hz. During the scan, all the participants were asked to keep their eyes closed but to stay awake and minimize head movements.

The rs-fMRI data were preprocessed via DPABI V7.0 (http://www.rfmri.org/dpabi) [65] and SPM12 (http://www.fil.ion.ucl.ac.uk/spm/software/spm12) via MATLAB R2022b. The preprocessing of the rs-fMRI data involved several steps: (a) the first 10 time points were discarded; (b) the remaining 440 time points were checked: the scanning time of each layer was inconsistent for scanner scans of the image layer by layer; the middle layer was used as a reference to correct the time difference of the other image layers; (c) spatial normalization was applied, and the layers were resampled to 3-mm isotropic voxel resolution; (d) spatial smoothing was performed; (e) the linear trend from the fMRI data was removed; and (f) nuisance covariates, including realignment correction, motion estimate regression, and the addition of head motion scrubbing regressors, were regressed out. Spikes were defined as volumes where framewise displacement exceeded 0.2 mm [66], and corresponding regressors were generated for these volumes. Subjects were excluded if their head motion exceeded 3 mm or 3° in any direction or if more than 10% of the volume was identified as spikes. In addition, according to the above exclusion criteria, no participant was excluded.

### Construction of edge-centric FBN

Faskowitz et al. [16] reported that edge time series (eTS) can be directly clustered, and the clustering results are highly similar to those derived from edge functional connectivity. Furthermore, the eTS method offers obvious advantages in computational efficiency, substantially reducing time and resource consumption. On the basis of these merits and previous studies, this study employs the eTS method to construct an edge-centric FBN [19]. The edge-centric FBN was constructed in 4 steps (Fig. 1A). First, the fMRI-blood oxygen level dependent (BOLD) signal of each brain region was acquired based on Schaefer200 brain template in the format of *N* × *T*, where *N* represents the number of brain regions and *T* represents the time points. Second, we calculated the values in pairs via the dot product of the time series for each brain region. From this calculation, we can form a new time series, called the eTS, which represents the magnitude of instantaneous co-fluctuations between pairs of brain regions. Third, we applied K-means clustering to the eTS and mapped the clustered eTS back to nodes to generate edge communities [19]. Finally, through edge communities mapped to brain regions, these edge communities naturally form widely overlapping node communities, called edge-centric functional brain networks, such that each node participates in multiple edge communities. In this study, we employed the K-means algorithm to divide the eTSs into 10 edge communities, and this process was repeated 25 times to mitigate the impact of random initialization [19]. The solution with the lowest within-community sum of squares was selected as the final community result [19]. In addition, to eliminate the influence of external factors such as sex, age, and years of education and to focus solely on the intrinsic patterns of BOLD signals, we performed a general linear model to remove the effects of sex, age, and years of education on the BOLD signals before construction of edge communities based on previous research [67,68]. To standardize the removal of individual and regional amplitude differences in BOLD signals, in this study, the *z* score method was applied to the BOLD signals after covariate removal analysis. The formula for the *z* score is as follows:

$$Z\left(t\right)=\frac{X\left(t\right)-\mu }{\sigma }$$ (1)

where *X*(*t*) is the signal value at time point *t*; *μ* is the mean of the time series; and *σ* is the standard deviation of the time series. In this study, the resulting edge community was expressed in a matrix, where the value signifies the community number obtained by K-means clustering, and the community number was determined by the edges linking brain regions *m* and *n*. The frequency of the edge community *s* in brain region *m* is described by Eq. 2 [69].

$$P_{\mathrm{ms}}=\frac{1}{N-1}\sum_{m\neq n}\delta \left(g_{\mathrm{mn}},\;s\right)$$ (2)

where *N* is the number of edge communities *s*; *g_mn_* is the edge’s community number linking brain regions *m* and *n*; and *δ*(*x,y*) is the Kronecker delta. In this study, the whole network was divided into 7 functional subnetworks: the visual network, somatomotor network, DAN, salience ventral attention network, limbic system, frontoparietal control network, and DMN [70]. These subnetworks mapped to the communities generated through K-means clustering, which provided a new perspective on the relationships between brain regions and systems within edge communities.

### Nodal entropy

The normalized entropy is the overlap and complexity in brain region *u* and is described by Eq. 3[16].

$$E_{u}=\frac{\sum_{s}\left(P_{\mathrm{ms}}.{\text{log}}_{2}P_{\mathrm{ms}}\right)}{{\text{log}}_{2}k}$$ (3)

where *p_ms_* is the probability of each subnetwork being in a certain community and where *k* is the maximum value of the subnetworks. In this study, we used normalized entropy as an index to measure the membership distribution of each brain region across communities. Higher nodal entropy corresponds to a more even distribution of brain regions in the community. Therefore, we can analyze the diversity and distribution of nodes via the entropy value.

### Classification analysis based on nodal entropy

We designed 2 classifiers for 2 distinct classification tasks: one for distinguishing between MDD patients and HCs and one for differentiating between MDDSI patients and MDDNSI patients (Fig. 1C). To address the issue of overfitting caused by excessively high feature dimensions, the RFE model was used to reduce the dimensionality of features of nodal entropy of each brain region. Then, a linear SVM was trained using nodal entropy; specifically, the SVM model utilized a linear kernel. All the parameters were optimized using a grid search approach on the training set to achieve the best classification performance. The dataset was divided into training and validation sets using a 5-fold cross-validation method. The classification performance of the 2 classifiers was evaluated on the basis of the average accuracy, average precision, average recall, and average F1 score across the 5-fold cross-validation. Additionally, after all the model training experiments, based on previous studies, we counted the selection frequency of each feature in all the experiments and selected the top 10 features with the highest frequency as the optimal feature subset [71].

### Connectome transcriptome association analysis

To explore the possible influence of genetic expression on nodal entropy, we obtained genetic expression data from the Allen Human Brain Atlas, which serves as a comprehensive resource to explore gene expression patterns across the human brain [72]. We performed a sequence of preprocessing actions using the abagen toolbox [73], which primarily consists of reannotating probes to genes, filtering data, selecting probes, assigning samples, and normalizing data. The brain region gene association matrix was the final output, where each entry represents the normalized expression level of a specific gene in a given brain region.

We employed partial least squares regression (PLSR) to examine the relationship between connectomics and transcriptomics [74]. In this PLSR model, the preprocessed gene expression matrix served as the predictor variable set, while the *t*-statistic values of the nodal entropy between MDDNSI and MDDSI groups derived from edge-centric networks served as the response variable set. We computed the first 15 PLS components and focused on the first component (PLS1) for our final analysis, as it captured the majority of the covariance between *X* and *Y* [75]. The significance of the PLSR model was determined using permutation tests that accounted for spatial autocorrelation adjustments. Moreover, a bootstrapping technique (number of iterations = 1,000) was used to evaluate and rectify the estimation errors associated with the weight contribution of each gene to the PLS1 component [76]. Ultimately, a ranked list of genes was compiled and highlighted those with significantly positive and negative weights as derived from the corrected weight values.

To gain insight into the biological significance of these significant genes, we performed enrichment analysis using KEGG, GO, and Reactome annotation databases via the Metascape platform [77]. The enriched terms were tested for statistical significance using a hypergeometric test, with *P* values adjusted for multiple comparisons via FDR correction (*P <* 0*.*05). We also performed a sensitivity analysis by varying the gene set size and comparing the consistency of enriched terms. The enrichment results revealed several overrepresented biological processes and pathways, such as synaptic transmission, neurotransmitter secretion, and axon guidance. The cell types are categorized into 7 classes based on spatial gene expression maps: astrocytes, endothelial cells, microglia, excitatory neurons, inhibitory neurons, and 2 groups of oligodendrocytes [78]. Since no significant enrichment was found in the PLS1+ gene set, the cell types in the PLS1− gene set were analyzed. The *P* value for the number of overlapping genes within each cell type was calculated using a permutation test.

### Statistical analyses

The analysis separated the 3 groups into the following 3 distinct components. (a) First, one-way analysis of variance (ANOVA) and chi-square tests were used to assess group differences in demographic characteristics, clinical characteristics, and cognitive performance. Two-sample *t*-tests were used to assess differences between MDDSI and MDDNSI groups in the total scores of HAMD-17 and third item scores of HAMD-17. In addition, we performed pairwise post-hoc tests for sex across the 3 groups using the chi-square test, with corrections applied using the Benjamini and Hochberg method. Additionally, we conducted post-hoc comparisons for age, years of education, and the 5 dimensions of the MCCB scale across the 3 groups via Tukey’s honestly significant difference method. (b) Initially, the Kruskal–Wallis test was employed to compare global and subnetwork entropy values among the 3 groups, as these continuous variables exhibited a nonnormal distribution. For entropy values showing significant differences according to the Kruskal–Wallis test, post-hoc comparisons between groups were conducted via the Mann–Whitney *U* test. To account for multiple comparisons, the FDR correction was applied to both the Kruskal–Wallis test and the post-hoc Mann–Whitney *U* test results. (c) Pearson correlation analyses were performed to examine the relationships between the subnetwork entropy values identified as significantly different in the previous step and cognitive performance or clinical characteristics. These correlations were calculated separately for the MDDNSI and MDDSI groups. FDR correction was also applied to adjust for multiple comparisons in the correlation analyses. The aforementioned methods were carried out via SPSS 24.0 (IBM Corporation). In addition, we define the significance levels as follows: *P < 0*.*05; **P < 0*.*01; ***P < 0*.*001.

## Data Availability

The access of the dataset presented in this paper is available on request to K.W. (kaiwu@scut.edu.cn). Code and example data for generating and analyzing edge time series are available in Github (https://github.com/guominxinxinxin/edge-centric-brain-functional).
