# Supplementary material for: Transcriptional Patterns of Nodal Entropy Abnormalities in Major Depressive Disorder Patients with and without Suicidal Ideation
Source: Research (Wash D C). 2025 Apr 2;8:0659. doi: 10.34133/research.0659 (PMC11964328; doi:10.34133/research.0659)
Supplement: Supplementary 1 — Fig. S1 Tables S1 to S6 [file research.0659.f1.docx]

**Supplementary Material**

**Title: Transcriptional patterns of brain entropy abnormalities in edge-centric brain functional networks in major depressive disorder patients with and without suicidal ideation**

Minxin Guo, Heng Zhang, Yuanyuan Huang, Yunheng Diao, Shixuan Feng, Jing Zhou, Yuping Ning, Fengchun Wu^*^, Kai Wu ^*^

**1. The post-hoc comparisons for demographic and clinical characteristics**

We performed pairwise post-hoc tests for sex across the three groups using the chi-square test, with corrections applied using the Benjamini & Hochberg method. Additionally, we conducted post-hoc comparisons for age, years of education, and the five dimensions of the MCCB scale across the three groups using the Tukey’s Honestly Significant Difference method. These results indicate significant differences in age, years of education, and the five dimensions scores of cognitive dimensions (except between MDDSI and MDDNSI), while sex differences were only significant between MDD patient groups (MDDNSI and MDDSI) and healthy controls, MCCB scores showed significant differences between patient groups and HCs but not within the MDD patient groups.

**2. Optimal Feature Selection Using Recursive Feature Elimination**

By employing recursive feature elimination (RFE), the SVM model can achieve optimal performance with a reduced feature set, enhancing interpretability and computational efficiency. The performance of the SVM model is evaluated by retaining different numbers of features, specifically from 20 to 200, in increments of 20. Hence, we examine 10 distinct feature subsets. For each subset, the SVM model is trained and validated using a five-fold cross-validation technique. The evaluation metrics recorded include accuracy, precision, recall, and F1-score. In this study, we designed two classifiers for distinct classification tasks: one for distinguishing between first-episode drug-naive major depressive disorder (MDD) patients and healthy controls, and the other for differentiating between MDD with suicidal ideation (MDDSI) and MDD without suicidal ideation (MDDNSI) patients. The averages and standard deviations of evaluation metrics of the classifier 1 and classifier 2 under different numbers of features are shown in **Table2 and Table 3**.

**Table 2.** The averages and standard deviations of evaluation metrics of the classifier 1 under different numbers of features

| Number of features | accuracy | precision | recall | F1-score |
| --- | --- | --- | --- | --- |
| 20 | 0.7324 ± 0.0428 | 0.7295 ± 0.0368 | 0.7500 ± 0.0298 | 0.7360 ± 0.0180 |
| 40 | 0.7277 ± 0.0491 | 0.7289 ± 0.0455 | 0.7400 ± 0.0400 | 0.7265 ± 0.0452 |
| **60** | **0.7578 ± 0.0460** | **0.7669 ± 0.0623** | **0.7600 ± 0.0499** | **0.7593 ± 0.0325** |
| 80 | 0.7277 ± 0.0593 | 0.7278 ± 0.0636 | 0.7400 ± 0.0533 | 0.7332 ± 0.0485 |
| 100 | 0.6978 ± 0.0735 | 0.7032 ± 0.0858 | 0.7100 ± 0.0558 | 0.7030 ± 0.0517 |
| 120 | 0.6773 ± 0.0722 | 0.6957 ± 0.0957 | 0.6600 ± 0.0712 | 0.6730 ± 0.0662 |
| 140 | 0.6472 ± 0.0964 | 0.6596 ± 0.1197 | 0.6600 ± 0.0573 | 0.6564 ± 0.0514 |
| 160 | 0.5762 ± 0.0815 | 0.5907 ± 0.0865 | 0.5600 ± 0.0573 | 0.5727 ± 0.0557 |
| 180 | 0.5309 ± 0.0725 | 0.5441 ± 0.0843 | 0.5300 ± 0.0909 | 0.5334 ± 0.0658 |
| 200 | 0.4951 ± 0.0887 | 0.5033 ± 0.0949 | 0.4700 ± 0.0772 | 0.4845 ± 0.0532 |

**Table 3.** The averages and standard deviations of evaluation metrics of the classifier 2 under different numbers of features

| Number of features | accuracy | precision | recall | F1-score |
| --- | --- | --- | --- | --- |
| 20 | 0.7043 ± 0.1659 | 0.7048 ± 0.2420 | 0.5872 ± 0.2862 | 0.6280 ± 0.2711 |
| 40 | 0.7360 ± 0.1611 | 0.7178 ± 0.2363 | 0.6526 ± 0.2970 | 0.6724 ± 0.2757 |
| **60** | **0.8187 ± 0.0973** | **0.8320 ± 0.0956** | **0.8000 ± 0.1546** | **0.8091 ± 0.1090** |
| 80 | 0.7773 ± 0.1058 | 0.7775 ± 0.0903 | 0.7667 ± 0.2198 | 0.7598 ± 0.1415 |
| 100 | 0.7197 ± 0.1238 | 0.7205 ± 0.1281 | 0.7000 ± 0.2392 | 0.6956 ± 0.1649 |
| 120 | 0.7193 ± 0.1652 | 0.6983 ± 0.1785 | 0.7129 ± 0.2624 | 0.6998 ± 0.2119 |
| 140 | 0.6213 ± 0.1098 | 0.6036 ± 0.1133 | 0.6192 ± 0.2237 | 0.6011 ± 0.1670 |
| 160 | 0.6463 ± 0.1125 | 0.6447 ± 0.1269 | 0.6359 ± 0.1746 | 0.6338 ± 0.1406 |
| 180 | 0.5480 ± 0.1271 | 0.5391 ± 0.1457 | 0.5038 ± 0.1725 | 0.5190 ± 0.1591 |
| 200 | 0.4990 ± 0.1050 | 0.4871 ± 0.1220 | 0.4718 ± 0.1525 | 0.4779 ± 0.1380 |

**3.** **Top ten contributive features for the classifier one identifying MDD patients and HCs**

**Table 4.** Top ten contributive features for the classifier one identifying MDD patients and HC

| Rank | regions | subnetwork |
| --- | --- | --- |
| 1 | RH_Vis_13 | visual network |
| 2 | RH_SomMot_8 | somatomotor network |
| 3 | RH_Limbic_OFC_2 | limbic system |
| 4 | RH_Limbic_TempPole_1 | limbic system |
| 5 | LH_SomMot_8 | somatomotor network |
| 6 | RH_Vis_11 | visual network |
| 7 | RH_Cont_pCun_1 | frontoparietal control network |
| 8 | LH_Limbic_TempPole_2 | limbic system |
| 9 | RH_Vis_10 | visual network |
| 10 | RH_SalVentAttn_FrOperIns_2 | salience ventral attention network |

**4. Top ten contributive features for the classifier one identifying MDDSI and MDDNSI**

**Table 5.** Top ten contributive features for the classifier one identifying MDDSI and MDDNSI

| Rank | regions | subnetwork |
| --- | --- | --- |
| 1 | LH_SomMot_12 | somatomotor network |
| 2 | RH_SalVentAttn_FrOperIns_2 | salience ventral attention network |
| 3 | RH_Default_PFCdPFCm_2 | default mode network |
| 4 | RH_SomMot_19 | somatomotor network |
| 5 | LH_SalVentAttn_Med_3 | salience ventral attention network |
| 6 | RH_Default_PFCdPFCm_1 | default mode network |
| 7 | RH_Default_PFCv_1 | default mode network |
| 8 | LH_DorsAttn_PrCv_1 | dorsal attention network |
| 9 | LH_DorsAttn_Post_7 | dorsal attention network |
| 10 | RH_DorsAttn_Post_6 | dorsal attention network |

**5. The correlation analysis results between top 10 features and scores from scales such as MCCB, HAMD-17, and BECK-SI**

Pearson correlation analyses were performed to examine the relationships between top 10 features and scores of scales in MDD group and MDDSI group. FDR correction was also applied to adjust for multiple comparisons in the correlation analyses. In addition, we define the significance levels as follows: *: *p* < 0.05; **: *p* < 0.01; ***: *p* < 0.001.

**Table 6.** The correlation between top 10 features and scores of scales in MDD group

|  | The total of HAMD-17 | Speed of Processing of MCCB | Attention/Vigilance of MCCB | Working Memory of MCCB | Verbal Learning of MCCB | Visual Learning of MCCB | Total score of MCCB |
| --- | --- | --- | --- | --- | --- | --- | --- |
| RH_Vis_13 | 0.069 | **-0.319**** | -0.169 | -0.12 | -0.098 | -0.137 | -0.238 |
| RH_SomMot_8 | 0.028 | 0.103 | -0.001 | 0 | -0.041 | 0.037 | 0.026 |
| RH_Limbic_OFC_2 | -0.12 | -0.097 | -0.195 | -0.025 | -0.088 | 0.01 | -0.115 |
| RH_Limbic_TempPole_1 | 0.067 | 0.184 | 0.072 | 0.096 | 0.037 | 0.097 | 0.136 |
| LH_SomMot_8 | 0.107 | -0.177 | -0.163 | -0.112 | -0.108 | -0.092 | -0.185 |
| RH_Vis_11 | 0.04 | -0.067 | -0.036 | -0.022 | 0.015 | 0.073 | -0.015 |
| RH_Cont_pCun_1 | 0.053 | 0.083 | -0.049 | 0.173 | -0.051 | 0.091 | 0.068 |
| LH_Limbic_TempPole_2 | 0.106 | -0.145 | 0.019 | 0.063 | 0.034 | -0.018 | -0.012 |
| RH_Vis_10 | -0.009 | 0.088 | 0.038 | 0.053 | 0.132 | 0.137 | 0.123 |
| RH_SalVentAttn_FrOperIns_2 | 0.12 | -0.063 | -0.028 | -0.02 | -0.069 | -0.075 | -0.07 |

**Table 7.** The correlation between top 10 features and scores of scales in MDDSI group

|  | The total of BSI-CV | The total of HAMD-17 | Speed of Processing of MCCB | Attention/Vigilance of MCCB | Working Memory of MCCB | Verbal Learning of MCCB | Visual Learning of MCCB | Total score of MCCB |
| --- | --- | --- | --- | --- | --- | --- | --- | --- |
| LH_SomMot_12 | 0.557 | 0.163 | 0.325 | 0.147 | 0.818 | 0.348 | 0.666 | 0.263 |
| RH_SalVentAttn_FrOperIns_2 | 0.01 | 0.84 | 0.299 | 0.281 | 0.82 | 0.42 | 0.802 | 0.412 |
| RH_Default_PFCdPFCm_2 | 0.725 | 0.58 | 0.638 | 0.528 | 0.597 | 0.574 | 0.923 | 0.767 |
| RH_SomMot_19 | 0.218 | 0.92 | 0.769 | 0.813 | 0.589 | 0.702 | 0.842 | 0.916 |
| LH_SalVentAttn_Med_3 | 0.242 | 0.437 | 0.678 | 0.716 | 0.343 | 0.474 | 0.351 | 0.359 |
| RH_Default_PFCdPFCm_1 | 0.279 | 0.385 | 0.559 | 0.335 | 0.964 | 0.623 | 0.106 | 0.544 |
| RH_Default_PFCv_1 | 0.913 | 0.502 | 0.959 | 0.338 | 0.527 | 0.976 | 0.997 | 0.939 |
| LH_DorsAttn_PrCv_1 | 0.88 | 0.277 | 0.966 | 0.999 | 0.012 | 0.237 | 0.065 | 0.141 |
| LH_DorsAttn_Post_7 | 0.042 | 0.985 | 0.543 | 0.137 | 0.157 | 0.366 | 0.69 | 0.178 |
| RH_DorsAttn_Post_6 | 0.865 | 0.771 | 0.288 | 0.054 | 0.293 | 0.362 | 0.298 | 0.1 |

**6.** **Transcriptional profiles associated with the SI**

The first PLSR component explained most of the total variance in regional topological differences between FDMDDSI and FDMDDNSI patients (**Figure. 1**).

**
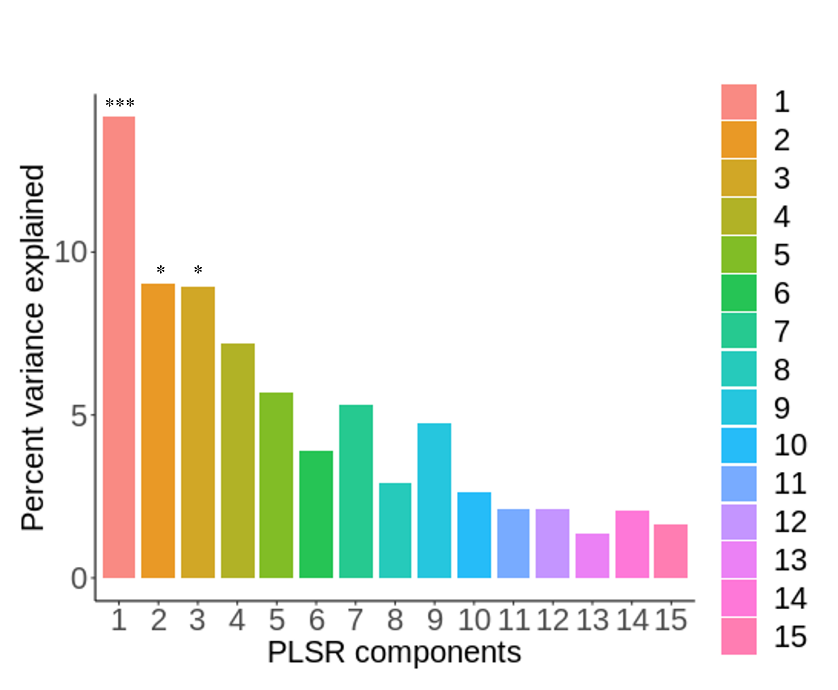
**

**Figure. 1** The percentage of explained variance for PLS components.
